# Fluid Balance in Surfers: A Scoping Review

**DOI:** 10.3390/sports14020049

**Published:** 2026-02-02

**Authors:** James Furness, Marie Troja, Abigail Brandon, Jasen Winny, Elisa F. D. Canetti, Kevin Kemp-Smith, Philip Abery, Gregory R. Cox

**Affiliations:** 1 Bond Institute of Health and Sport, Faculty of Health Sciences and Medicine, Physiotherapy Department, Bond University, Robina, QLD 4226, Australia; jfurness@bond.edu.au (J.F.); ecanetti@bond.edu.au (E.F.D.C.); kkempsmi@bond.edu.au (K.K.-S.); pabery@bond.edu.au (P.A.); 2 Bond Institute of Health and Sport, Faculty of Health Sciences and Medicine, Nutrition and Dietetics Department, Bond University, Robina, QLD 4226, Australia

**Keywords:** water sports, dehydration, fluid loss, hydration status, wetsuits

## Abstract

Surfing, performed semi-submerged in varied environmental conditions and apparel, evokes unique fluid losses compared to land-based sports, despite the inherent difficulties in collecting urine losses in surfing, making direct sweat loss comparisons challenging. This scoping review aimed to identify factors that influence fluid balance in surfing. Nine databases were systematically searched for relevant studies specific to the concept of fluid balance in surfing participants. A total of 153 studies were screened, and seven met the inclusion criteria. Five studies were cross-sectional designs, and two were randomised controlled trials, aligning to levels of evidence IV and II, respectively. Reported body mass loss was 1.3%, and fluid loss was 0.57 L/h. The surfers wearing wetsuits incurred a greater decrease in body mass following a surf session (1.05% vs. 0.59%, respectively). Elite surfers wearing wetsuits were reported to have high fluid losses (1.68 L/h), resulting in a body mass loss of 3.9%. Reported fluid losses of surfers are lower than those of land-based athletes; however, the elite surfers were reported to have high fluid losses that would likely impede exercise performance. Available information on fluid losses in surfers is of poor quality; thus, further research is needed to elucidate fluid intake recommendations for surfers in varying environmental conditions and wearing different surf apparel.

## 1. Introduction

Water is a fundamental component of the human body, constituting approximately 50–60% of total body mass [1]. Physical activity alters body water content via a variety of physiological responses essential to maintaining homeostasis and regulating body temperature [1]. When engaging in physical activity, muscle contraction is the primary contributor to an increase in body temperature [2], ultimately initiating a sweating response [1,3]. Continuous, vigorous exercise will lead to excessive sweating and result in dehydration if adequate fluid is not consumed [4]. Dehydration has detrimental effects on cognitive function [1,3,5], strength, endurance and fatigue perception [6]. It is well accepted that body mass losses during exercise should be minimised to less than 2% to preserve physical and cognitive performance [3,7,8]. 

Factors such as weather conditions including ambient temperature, the type of sports apparel, intensity and the duration of physical activity influence the sweating response [3,9]. During land-based sports, thermoregulation responses rely primarily on evaporative heat exchange [3]. This mechanism encompasses the evaporation of sweat from the air movement across the skin [1,3]. However, individuals performing aquatic-based activities show unique features in terms of thermoregulation. During physical activity performed in water, whether it is in a pool, ocean or lake, body core temperature tends to be lower [7], and skin temperature becomes congruent with the surrounding water temperature [10,11]. This difference is attributed to increased forced convective and conductive heat transfer from the skin [7,12]. Research on masters swimmers has demonstrated that elevated sweat losses occur in warmer water conditions and at greater intensities of physical activity [13]. Unlike swimming and many other aquatic sports, surfers are semi-submerged in water, so they are likely to incur unique fluid losses.

Due to its unique nature, surfing presents distinct challenges to accessing and consuming fluids compared to other aquatic-based activities such as pool-based swimming, synchronised swimming and open-water swimming. Pool-based swimmers typically perform for short periods of time and have access to fluids at the end of the pool, whereas open water swimmers are typically supported from feeding pontoons or individual feed boats to drink during exercise [7]. Surfers commonly surf for extended periods of time, from 20–30 min for competitive events to 4–5 h as a recreational activity, with no access to fluids while in the water [14]. Environmental conditions (size of the waves, wind direction and speed, tides, water temperature) generally dictate the time surfers spend in the water [14]. The time spent in the water surfing is marked by distinct phases, including paddling (constituting approximately 55% of the time), stationary intervals (approximately 34%) and wave riding (approximately 3–8%) [14,15]. In addition, surfers can wear different surf apparel (from boardshorts to whole-body wetsuits), which can affect thermoregulatory processes and, consequently, fluid losses [16].

Previous studies investigating fluid losses of surfers have reported that extended surf sessions result in a body mass loss, reflective of fluid losses that may decrease surfing performance [10,17,18,19]. Studies have also reported that water temperature and surf apparel may affect fluid losses in surfers [10,18,19]. Nevertheless, to the best of the authors’ knowledge, no review has been conducted to report fluid balance of surfers. The substantial growth of surfing over the last two decades, with a current estimate of 37 million surfers world-wide [20] and its recent inclusion into the 2021 Summer Olympic Games [21] warrants investigation into factors that influence fluid balance of surfers to assist in the development of surfing specific fluid intake guidelines. Given the absence of previous literature reviews and the variation in types of literature, a scoping review is the most suitable design at this stage. Therefore, this scoping review was guided by the following research question: how does surfing influence fluid balance?

The scoping review was underpinned by the following objectives:

(1) Evaluate the volume and type of scientific literature on surfing and fluid balance;
(2) Provide a summary of studies reporting fluid balance and hydration status of surfers;
(3) Determine the influence of apparel (wetsuits) on fluid balance of surfers;
(4) Identify knowledge gaps and areas for future research.

## 2. Materials and Methods

### 2.1. Protocol and Registration

A scoping review was adopted to address the broad nature of both the research question and associated objectives. A protocol was developed using the Preferred Reporting Items for Systematic Reviews and Meta-Analyses (PRISMA) Extension for Scoping Reviews (PRISMA-ScR) checklist [22], see Supplementary File S1. The finalised protocol was registered with Open Science Framework (https://doi.org/10.17605/OSF.IO/7CTPJ, accessed on 29 September 2023).

### 2.2. Eligibility Criteria

This study’s eligibility criteria were shaped using the Joanna Briggs Institute’s recommended Population, Context, Concept (PCC) framework model [23].

### 2.3. Population

The study population consisted of male and female surfers of any age, encompassing both elite and recreational levels of participation, defined through the participant classification framework [24]. Elite surfers included those that were competing at the national or international level or those highly proficient in skills required to perform the sport; for example, professional surfers that may or may not compete were considered elite. Recreational surfers were defined as surfers who participate in surfing but not at a competitive local level or above. Additionally, this review considered all types of surf apparel, including wetsuits, rash guards, boardshorts, bikinis and other relevant attire. Any studies that included participants with health conditions that would affect thermoregulation and surf fitness (e.g., cardiovascular disease, pulmonary disease and kidney conditions) were excluded.

### 2.4. Concepts

The concept of this scoping review was hydration and fluid balance in surfing participants, which involved fluid balance metrics (e.g., fluid losses) and hydration metrics (e.g., body mass change in kilograms or as percentage, urine specific gravity and osmolality).

### 2.5. Context

Studies performed in all geographical locations, and a variety of environments were included. No time limit was established for the inclusion of studies. Furthermore, all study designs that reported on fluid intakes and outputs were included in this review. Due to the broad nature of a scoping review design, this study included conference abstracts, posters and reports from governing bodies (e.g., World Surf League, Surfing Australia); magazine articles were excluded. Studies in English, French, Spanish and Portuguese were included.

### 2.6. Information Sources and Search Strategy

A stepwise approach was employed for gathering relevant articles for this scoping review. First, an initial search on PubMed was conducted to analyse keywords in titles and abstracts to create an initial key terms list. After consulting with four authors (AB, MT, JF and JW), additional key terms were included and validated using relevant PubMed reference numbers (PMIDs) through the Systematic Review Accelerator (SRA): Search Refinery [25]. Secondly, a comprehensive PubMed search strategy was developed in collaboration with the University librarian, who is skilled in advanced search strategies. Additional keywords, Medical Subject Headings (MeSHs), and Title–Abstract (TIAB) search field tags were added to the search strategy.

The PubMed search was then converted into a compatible format for all other databases using SRA: The Polyglot Search Translator [26]. A modified search was utilised to identify non-indexed literature in Google Scholar. Finally, all key reference lists of selected studies were searched for relevant articles. See Supplementary File S2 for search strategies utilised for the selected databases. The search was initially conducted in September 2023 and then again in July of 2024 and in November 2025, with no new relevant articles identified in the second or third searches.

### 2.7. Selection of Sources of Evidence

All identified articles were retrieved from PubMed, Embase, CINAHL, SPORTDiscus, Web of Science, Scopus, ProQuest Health and Medical Collection, ProQuest Dissertations and Thesis Global, and the first 100 results from Google Scholar, as per CADTH Grey Matters: A Practical Search Tool for Evidence Based Medicine [27]. These articles were imported into Endnote, where duplicates were removed [28]. Screening by title and abstract was then independently conducted by two authors (MT and JW), to identify articles for inclusion in a full-text review. A consensus process was undertaken by two authors (MT and JW), by comparing Endnote libraries, and any disagreements were resolved with a third author (AB). The articles were retrieved in full text, and any non-English articles were translated using the translating tool DeepL Translator [29] and then crosschecked by MT, an author fluent in Portuguese, Spanish and French. Two authors (MT and JW) independently applied the inclusion and exclusion criteria to the full-text articles, and any discrepancies were resolved by a third author (AB) to reach a consensus on which articles to include in this scoping review.

### 2.8. Data Charting and Data Items

An initial data extraction table was created in an Excel spreadsheet and was pre-piloted, in accordance with the Joanna Briggs Institute (JBI) Methodology Guidance for Scoping Reviews [23]. Subsequently, data from each article was collaboratively documented, and the table underwent modifications and adaptations, as key data points became apparent during the review process.

The final data extraction table encompassed the following elements: authors, title, year of publication, aim/purpose, type of publication/location of publication, study design, level of evidence, population, recruitment processes, location/context, methods, outcome measures/measurement tool, hydration and fluid balance measures (such as pre and post body mass), statistical results and summary of results. Two authors (AB and JW) graded the level of evidence for each included study according to the Australian National Health and Medical Research Council (NHMRC) criteria [30]; disagreements were resolved through discussion with a third author (MT). This process was used to describe the study rather than a formal evaluation process involving critical appraisal. Contact was made with the co-authors from two studies [10,31] to obtain the means and standard deviations of the pre- and post-surf session body mass for participants. Atencio et al. [10] provided data on the apparel worn (wetsuit) in the locations of the Gold Coast and San Diego. This enabled researchers to extract and present data for both wetsuit and no-wetsuit conditions in both locations.

### 2.9. Data Synthesis

Data items from the included studies have been synthesised and presented in tables with accompanying narrative syntheses. To allow for synthesis of results across multiple studies, commonly used hydration measures have been presented and calculated, such as body mass change (absolute and relative) and fluid loss (absolute and hourly rate). Body mass change in kilograms (kg) was calculated by subtracting the pre-exercise body mass from the post-exercise body mass. A relative body mass change expressed as a percentage (%) was calculated using the following formula:

$$Percent\;change\;in\;body\;mass\;\left(\%\right)=\left\{\frac{\left(pre\text{-}exercise\;body\;mass\left(kg\right)-post\text{-}exercise\;body\;mass\left(kg\right)\right)}{pre\text{-}exercise\;body\;mass\left(kg\right)}\right\}\times 100$$

Studies either failed to capture urine loss during surfing or failed to mention whether this information was collected. It is likely that participants in the included studies urinated when surfing, so we have reported fluid loss as opposed to sweat loss. Fluid loss (L) and fluid loss rate (L/h) were calculated using the following formula:

$$Fluid\;Loss\;\left(L\right)=\;\left(pre\text{-}exercise\;body\;mass\;\left(kg\right)-post\text{-}exercise\;body\;mass\;(kg)\right)+fluid\;intake\;Fluid\;Loss\;Rate\;\left(L/h\right)=\;\frac{\left(pre\text{-}exercise\;body\;mass\;\left(kg\right)-post\text{-}exercise\;body\;mass\;(kg)\right)+fluid\;intake}{Duration\;(h)}$$

A subsequent calculation of mean body mass change (kg) across multiple studies was also calculated using Microsoft Excel. To account for varying sample sizes, a weighted mean was also calculated using the formula:

$$\frac{\sum \left(w_{i}\cdot x_{i}\right)}{\sum w_{i}}$$

where

- $w_{i}$ represents the sample size of each study.
- $x_{i}$ represents the body mass change from each individual study.

The results were also presented to examine the differences in relative body mass (%) change and fluid loss rate (L/h) between participants wearing wetsuits and those not wearing them.

## 3. Results

### 3.1. Sources of Evidence

**Figure 1:**
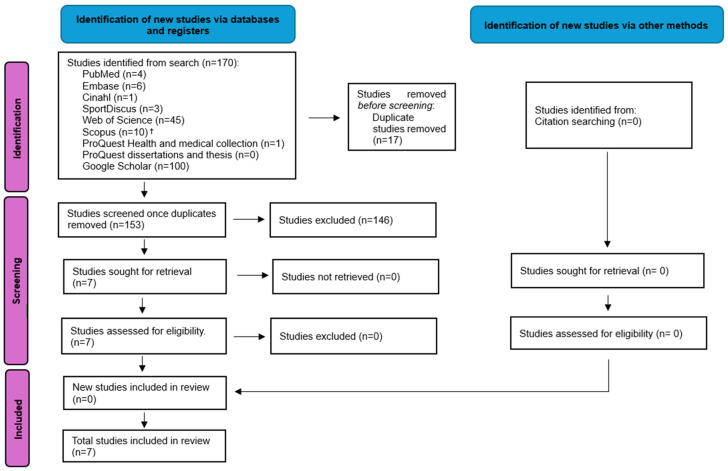
PRISMA flow diagram of the record identification process [22]. ^†^ One additional record was identified when the search was completed again in July 2024.

A combined total of 170 records were obtained from the database searches and after the removal of duplicates, 153 records were screened by title and abstract, as per the eligibility criteria, resulting in the exclusion of 146 records. Full-text versions of the remaining seven records were screened and met the inclusion criteria for this scoping review. Two authors (JW and MT) conducted citation searching of the included full-text articles, and no additional articles were included. The PRISMA flow diagram in Figure 1 illustrates the selection of sources of evidence [22].

### 3.2. Study Characteristics

A summary of the study characteristics is presented in Table 1. In alignment with the NHMRC levels of evidence [32], level IV evidence using an observational cross-sectional design was most common (n = 5) [10,18,19,31,33], followed by level II evidence using randomised controlled trial design (n = 2) [17,34].

### 3.3. Publication Type and Year of Publication

Amongst the seven studies included, five were journal articles [10,18,19,31,34], one was a conference abstract [33], and one was a thesis [17]. Two studies were published in 2008 and 2009 [17,34], three were published between 2015 and 2019 [18,19,33], with the remaining two studies published in 2021 [10,31].

### 3.4. Location of Data Collection

The majority of studies were conducted in Australia (Northern New South Wales [18,19,31] and Sydney [17]). One study collected data from three different locations, including the Gold Coast, Australia, Costa Rica and San Diego, USA [10]. Two studies were completed in Brazil, at Praia Mole de Florianopolis [34], and Sao Paulo [33].

### 3.5. Population Characteristics

All included studies reported age, gender and surfing level, with six out of seven reporting body mass. The average age was 27.0 years, with six out of seven studies including males only [17,18,19,31,33,34]. The study by Atencio et.al., [10], included 254 male and 52 female surfers. Most participants in the studies were recreational surfers, with two studies [17,33] including elite (professional) surfers.

### 3.6. Summary of Outcome Measures

A range of outcome measures were used to assess the impact of surfing on fluid balance and hydration status (Table 2) [10,17,18,19,31,33,34]. The most common outcome was determining fluid balance through assessing body mass changes (both absolute and relative) pre- and post-surfing [10,17,18,19,31,34]. Urinary biomarkers were assessed using a digital refractometer, including urine specific gravity (USG) [17,31]. Pre- and post-urine osmolality and colour were collected in two studies [18,19]. Urine colour was determined by comparing the collected urine against the NCAA’s Assess Your Hydration Status urine colour chart [35]. One study [33] collected blood samples, including haematocrit (Ht), sodium (Na), potassium (K), urea and creatine kinase, before and after sessions and 12 h after a resting period. All studies quantified the surf session length, and two studies further quantified the session using the Global Positioning System (GPS) [31,33]. A range of environmental outcomes were also reported, with water temperature (°C) and ambient temperature (°C) being most reported [10,17,18,19,31,34]. Only two studies collected heart rate data from the participants [10,31].

### 3.7. Type of Apparel

Six of the seven studies reported on the type of apparel used, which included boardshorts, rash shirts and wetsuits [10,17,18,19,31,34]. All apparel types are outlined in Table 3. The choice of surf apparel was influenced by environmental factors, specifically water temperature, with participants wearing board shorts in water temperatures ranging from 24.2 to 28.6 °C [10,18,19,31] and wetsuits in water temperatures ranging from 16.8 to 24.9 °C [10,17,18,34]. No included studies specifically examined the influence of apparel on body mass change.

### 3.8. Duration of Exposure

All seven studies reported the duration of surf sessions in minutes (Table 3). The duration ranged from 60 min to 120 min. Six of the studies completed only one session [10,17,18,19,31,34], whereas Burini et al. [33] conducted three 90 min sessions within a 12 h period.

### 3.9. Intervention Exposure

Two studies [17,34] incorporated intervention groups to assess the effect of fluid intake on hydration status (i.e., body mass change). Carrasco [17] allocated participants into two groups: one group received water (3 mL/kg body mass) every 20 min during the 100 min surf session, while the other group acted as a control (no fluids were provided). Unsurprisingly, the group of surfers that consumed water had a significantly lower mean body mass loss (1.6 ± 0.7%) compared to the control group (3.9 ± 0.7%; see Table 3).

The second intervention study conducted by Somensi [34] implemented three distinct interventions for fluid intake, with each group consuming 500 mL of fluids 20 min before starting the surfing session. Subsequently, during the surfing session, the control group consumed water, while the intervention groups consumed either a formulated drink (6% carbohydrate; 0.7 g/L sodium) or a commercial carbohydrate–electrolyte solution (6% carbohydrate; 0.45 g/L sodium). The control group recorded the largest body mass loss 0.98% (SD 0.14), whilst the surfers consuming the formulated drink and commercial carbohydrate-electrolyte drink incurred a body mass loss of 0.63% (SD 0.66) and 0.70% (SD 0.84), respectively.

### 3.10. Results of Individual Sources of Evidence

#### 3.10.1. Fluid Balance and Fluid Loss

Table 3 presents the fluid balance results for all studies. This table includes calculations for body mass change as an absolute and relative value. In addition to this, fluid loss rate was calculated; however, this is not a substitute for sweat loss rate. The relative body mass losses ranged from 0.5 to 3.9%, with an average loss of 1.3% and a weighted average loss of 0.89%. The average fluid loss rate was 0.57 L/h, and the weighted average was 0.54 L/h. A sub-analysis was conducted of the observational cross-sectional studies to investigate the impact of a wetsuit on body mass losses (see Table 4). For recreational surfers, average body mass and fluid loss rate were lower for wetsuit trials (0.59% and 0.37 L/h, respectively) compared to no-wetsuit trials (1.05% and 0.50 L/h, respectively). For elite surfers, body mass loss and fluid loss rate were threefold higher (3.90% and 1.7 L/h, respectively) compared to the data reported for recreational surfers (Table 4).

#### 3.10.2. Urinary Biomarkers

Two studies were conducted pre- and post-USG measures. Carrasco [17] reported an increase from 1.007 to 1.029, with a smaller increase in the fluid intake group (1.009 to 1.014). Conversely O’Neill et.al. [31] reported a higher pre-trial USG of 1.017, which decreased to 1.015 at the end of the surf session. Urine osmolality measured in two studies [18,19] decreased from 668.6 to 632.9 mOsmol/kgH_2_O when not wearing a wetsuit, compared with 600.0 to 495.7 mOsmol/kgH_2_O when wearing a wetsuit, indicating an increase in urine concentration.

### 3.11. Standardisation Techniques for the Assessment of Body Mass Change

As outlined in the method section, studies failed to account for urine loss, and hence sweat loss rate could not be explicitly determined. For this reason, the outcome fluid loss rate was used. Table 5 characterises standardisation techniques undertaken in included studies that could influence measurement of body mass change in participants engaging in aquatic sports. As documented above elite surfers’ body mass loss and fluid loss rate were three-fold higher than recreational surfers. The only study to include elite surfers, [17], provided participants with 1000 mL of water the night prior to the trial and did not account for urine loss during the trial.

## 4. Discussion

This scoping review was guided by the research question “How does surfing influence fluid balance?” Despite an extensive search of the literature only seven studies met the inclusion criteria for this scoping review. Fluid losses and changes in body mass were 0.57 L/h and −1.3%, respectively, which appear lower than those commonly reported in land-based athletes [36]. Elite status, wetsuit use, duration and water temperature appear to be the primary factors influencing differences in reported fluid losses in surfers and requires further investigation. As illustrated within the review, there is heterogeneity in the designs, methods, outcome measures and findings. To enhance clarity where possible findings will be discussed where there was commonality between studies. Furthermore, a central limitation of studies on surfing is being able to distinguish sweat losses from fluid losses due to the inability to collect urine losses during surfing sessions. As such, we reported fluid losses as opposed to sweat losses, which are most commonly reported in land-based sports. This makes direct comparisons with land-based sports difficult. An appreciation of this subtle difference needs to be considered when reviewing these comparisons throughout the discussion.

### 4.1. Volume and Type of Scientific Literature on Surfing and Fluid Balance

The first objective of this scoping review was to evaluate the volume and type of scientific literature on surfing and hydration. The majority of the included studies (five of seven) were cross-sectional designs [10,18,19,31,33], and two were randomised controlled trials [17,34], aligning with NHMRC [30] level IV and II, respectively. Not only was a small quantity of data identified, but a low level of evidence was highlighted. These results strengthen the requirement for further research in the field of hydration and fluid losses in surfing.

### 4.2. Summary of Studies Reporting Fluid Losses and Hydration Status of Surfers

The primary outcome used in most of the included studies was fluid loss (L/h) and body mass change (%) in response to a surf session. Fluid loss rather than sweat loss was reported, as studies failed to account for urine losses during surf sessions. The characteristics of the surfers and environmental parameters were quantified, which allowed for comparisons and synthesis of findings from this review. Overall, the fluid losses of surfers were 0.57 L/h, and the average percent change in body mass was 1.3%. In comparison, normative data comprising of 1303 recreational and competitive participants from a range of land-based sports (basketball, soccer, American football) reported an average sweat rate of 1.13 L/h, with American footballers having the greatest sweat losses (1.51 L/h) [36]. Reported body mass losses of land-based sport athletes [37] appear higher than those of recreational surfers, despite limited or no access to fluid intake when surfing. This suggests that surfers are better able to maintain hydration status, principally due to a lower sweat rate as access to fluid intake while surfing is limited.

Fluid losses of surfers appear similar to reported fluid losses of other aquatic sport athletes such as water polo players (0.29 L/h) [38] and swimmers (0.31 L/h) [39]. When immersed in water, the capacity for heat dissipation is increased due to a higher forced convective and conductive heat transfer from the skin [12]. Subsequently, body core temperature is lower, resulting in decreased sweat losses [40]. However, water absorbed through the skin via passive diffusion may contribute to an underestimation of fluid losses in aquatic sport athletes as they are submerged or semi-submerged throughout exercise [41].

### 4.3. The Influence of Apparel (Wetsuits) and Surfing Level on Fluid Balance in Surfers

Reported fluid losses in recreational surfers within included studies was similar irrespective of surf apparel worn with surfers wearing a wetsuit incurring an average fluid loss of 0.37 L/h compared to surfers not wearing a wetsuit of 0.50 L/h. Despite similar hourly fluid losses, body mass loss was almost double in surfers not wearing a wetsuit (1.05%), compared to surfers wearing a wetsuit (0.59%). The average water temperature for the recreational surfers wearing a wetsuit within included studies was 19 °C compared to 26 °C for surfers wearing a rash shirt (for sun protection) or board shorts only. The thermoregulatory response in surfers wearing either a rash shirt or board shorts in warmer water likely differs to that of surfers wearing a wetsuit in cooler water, resulting in greater fluid losses [31]. Further, cooler water temperatures may facilitate heat loss due to higher convective properties [42], subsequently reducing fluid losses. Macaluso et al. [13] further verified this thermoregulatory response in swimmers; demonstrating that warmer water temperatures resulted in an increase in body mass loss. Furthermore, warmer water temperatures have been shown to result in greater cardiovascular demands with higher heart rates when comparing the same exercise in cooler water temperatures [43]. The mechanisms behind these cardiovascular demands involve the need to dissipate heat through the skin; smooth muscle relaxation increases vascular vasodilation and consequently leads to higher heart rates [44].

In contrast to the reported fluid losses for recreational surfers, a study by Carrasco [17] reported a body mass change of 3.9% and hourly fluid losses of 1.68 L/h in a group of professional surfers during an extended session in cooler water. It is likely that the higher fluid losses observed in this elite cohort of surfers is due to the intensity of exercise undertaken. Competitive surfers spend more time paddling (~54%) during competition [45] compared to recreational surfers (~44%) during a surf session [46]. Not surprising, heart rates (average and peak heart rates) have been shown to be higher for competitive surfers (140–190 bpm) [45], compared with recreational surfers (135–171 bpm) [46]. While the study by Carrasco [17] was not a competitive event, the surfer’s performance was judged on the first and last 20 min of the 100 min session, creating a competitive simulation.

Notwithstanding that the differences in intensity of exercise between elite and recreational surfers may explain the differences in reported fluid losses observed in included studies, it is imperative to consider the pre-trial standardisation strategies employed by Carrasco [17] when interpreting these findings. Participants were required to drink 1 L of water before sleep the night before the trial. The following morning participants presented hyperhydrated [47], with an average USG score of 1.007. While this strategy standardised pre-trial hydration status, it likely increased urine losses in the subsequent surf session, thus increasing reported fluid losses. Antidiuretic hormone (ADH) is not suppressed in water, which would further exaggerate fluid losses in this study. The increased metabolic demand in response to the competitive simulation may provide some explanation of the higher reported fluid losses in elite surfers [17]. However, the pre-trial methodology likely increased urine losses and subsequent fluid losses reported. As such, these results should be interpreted cautiously and require further research for confirmation.

### 4.4. Identify Knowledge Gaps and Areas for Future Research

The included studies provide a profile of the influence of surfing on fluid loss and body mass loss of surfers. However, due to methodological challenges, included studies within this scoping review failed to identify the contribution of sweat and urine loss to overall fluid loss in surfers. Future research should carefully consider the logistics of data collection in the field to capture urine losses of surfers, in order to more clearly understand sweat losses of recreational and competitive surfers. Further, there is a lack of understanding regarding the influence of water temperature, surf apparel (namely wetsuits), surf session intensity and duration on fluid losses of surfers. Given the stark differences in observed fluid losses on elite surfers compared to recreational surfers in included studies, there is a clear need for further research specific to this group of surfers.

### 4.5. Limitations

This review is not without limitations. Despite our comprehensive review of the literature, a limited number of eligible studies met the inclusion criteria. The authors acknowledge that confidence in conclusions may be limited due to the inability of included studies to address confounding variables (such as urine losses and lack of pre-trial standardisation; Table 5). Given the heterogeneity of the designs, apparel, water temperature, duration and outcome measures, the presented results should be seen as descriptive and should not be interpreted as normative benchmarks for surfers’ fluid losses.

### 4.6. Strengths

A primary strength of this scoping review is the comprehensive search methodology used across nine databases. Moreover, language and country of study bias were addressed by including research in multiple languages. Furthermore, the reduction in selection bias was supported by the involvement of two researchers in the initial screening process, with any discrepancies resolved by a third researcher. Lastly, the review eligibility criteria, encompassing all types of study designs, minimised the potential for selection quality bias.

## 5. Conclusions

Reported fluid losses of surfers are similar to other aquatic sport athletes and appear lower than land-based athletes, most likely due to different thermoregulatory responses when submerged or semi-submerged in water. Body mass losses reported in elite surfers during an extended competition scenario were greater than reported losses of recreational surfers. However, data on elite surfers are from a single study and pre-trial hyperhydration, and competitive simulation likely inflated fluid loss estimates reported. Therefore, these results should be considered cautiously and require further research. Finally, while some attempts have been made to understand the interaction between intensity of surfing, water temperature and surf apparel, the implications of fluid losses of surfers are unclear and warrant future investigation.

Practical Implications:

Based on the available evidence included in this scoping review, the following practical applications should be considered with caution.

1. There is no requirement for fluid intake while surfing for 1–2 h in water temperatures ranging from 19 to 26 °C in recreational surfers in order to maintain hydration status.
2. Elite surfers wearing a wetsuit may incur high fluid losses during extended, strenuous surf sessions and should plan to consume additional fluids throughout the session.
3. When assessing fluid balance during exercise to inform fluid and sweat losses in surfers, practitioners and researchers should standardise assessment techniques and consider strategies to capture urine losses.

## Figures and Tables

**Table 1:** Study characteristics of included studies [10,17,18,19,31,33,34].

	Level of Evidence	Number of Studies	References
Study Design			
Observational cross-sectional	IV	5	[10,18,19,31,33]
Randomised controlled trial	II	2	[17,34]
Publication Types			
Thesis		1	[17]
Conference abstract		1	[33]
Journal		5	[10,18,19,31,34]
Year of Publication			
2021		2	[10,31]
2019		1	[18]
2017		1	[33]
2015		1	[19]
2009		1	[34]
2008		1	[17]
Location of Data Collection			
Australia		5	[10,17,18,19,31]
Brazil		2	[33,34]
Costa Rica		1	[10]
United States		1	[10]
Population Gender			
Male		7	[10,17,18,19,31,33,34]
Female		1	[10]
Surfing Level			
Recreational		5	[10,18,19,31,34]
Elite		2	[17,33]
Duration of observation (Minutes)			
90–120		3	[17,31,33] *
70–86		3	[10,18,19] **
60–70		1	[34] *

* Set duration of surf session by researchers. ** No set duration of surf session by researcher.

**Table 2:** Outcome measures reported in included studies [10,17,18,19,31,33,34].

	Fluid Balance		Urine and Blood Parameters				Physiological Parameters		Surf Session		Environmental Parameters			
Study	BM Change (kg)	BM Change (%)	UC *	USG	UO (mOsmol/kgH_2_O)	Blood Samples †	Tympanic Temp (°C)	HR (bpm)	Surf Length (min)	GPS	Surf Conditions ††	Relative Humidity (%)	Ambient Temp (°C)	Water Temp (°C)
Atencio et al. [10]	x	x						x	x		x		x	x
Burini et al. [33]						x			x	x				
Carrasco [17]	x	x		x					x		x		x	x
Meir et al. [19]	x	x	x		x		x		x		x	x	x	x
Meir et al. [18]	x		x		x		x		x		x	x	x	x
O’Neill et al. [31]	x	x		x				x	x	x	x		x	x
Somensi et al. [34]	x	x							x				x	x

* NCAA Assess Your Hydration Status Colour Chart; x = reported outcome measure; BM = body mass; UC = urine colour; USG = urine specific gravity; HR = heart rate, GPS = Global Positioning System. † Blood samples were collected for haematocrit (Ht), sodium (Na), potassium (K), urea and creatine kinase (CK) levels before, after sessions and 12 h after the resting period. †† Surf conditions such as swell size, direction and period.

**Table 3:** Main findings shared across multiple articles [10,17,18,19,31,33,34].

	Participant Characteristics		Study Characteristics		Environmental	Urinary Biomarkers	Fluid Balance		
Study	Age (Years) Surfing Level	Apparel (n)	Duration (min)	Location (Type of Break)	Water Temp (°C)		Body Mass Δ (%)	Body Mass Δ (kg)	Fluid Loss Rate (L/h)
Atencio et al. [10]	34.4 (10.5) Recreational	Skin or Rash Guard (n = 158)	83 (34)	Costa Rica (beach break)	28.6 (0.9)		−0.98 (0.78)	−0.72 (0.60)	0.52
		Skin or Rash Guard or Wetsuit ≤ 3 mm (n = 52)		Gold Coast, AUS (beach break)	24.9 (1.0) º	NR	−0.67 (0.46)	−0.94 (0.66)	0.68
		Wetsuit ≤ 3 mm or Wetsuit ≥ 3 mm (n = 96)		San Diego, USA (beach break)	16.8 (1.8) º		−0.48 (0.55)	−0.36 (0.43)	0.26
Burini et al. [33]	24.0 (1.0) Elite	NR (n = 6)	3 × 90 min (specified time)	Brazil (NR)	NR	NR	NR	NR	NR
Carrasco [17]	27.0 (3.3) Elite	Wetsuit (≤3 mm) (n = 12)	100 (specified time)	Sydney, AUS (beach break)	20.8 (1.4)	µ Pre_NF_: 1.007 (0.004)	−3.9 (0.7)	−2.8 (0.6)	1.68
						µ Post_NF_: 1.029 (0.003)			
						µ Pre_FI_: 1.009 (1.005)	−1.6 (0.7) ^†^	−1.1 (0.4) ^†^	1.32 ^ꞩ^
						µ Post_FI_: 1.014 (0.005)			
Meir et al. [19]	21.6 (2.5) Recreational	Skin or Rash Guard (n = 7)	76 (6)	Northern NSW, AUS (beach break)	24.3 (0.8)	Ò Pre: 668.6 (184.9)	−1.2 (1.6)	−0.9 (1.2)	0.72
						Ò Post: 632.9 (291.2)			
Meir et al. [18]	23.0 (2.8) Recreational	Skin or Rash Guard (n = 7)	76 (5)	Northern NSW, AUS (beach break)	24.3 (0.8)	Ò Pre: 668.6 (184.9)	−1.2 *	−0.9 *	0.72
						Ò Post: 632.9 (291.2)			
		Wetsuit (≤3 mm) (n = 7)			20.0 (0.0)	Ò Pre: 600.0 (268.2)	−0.76 *	−0.60 *	0.48
						Ò Post: 495.7 (220.1)			
O’Neill et al. [31]	33.20 (5.9) Recreational	Skin or Rash Guard or Neoprene Rash vest (n = 10)	120 (specified time)	Gold Coast, AUS (beach break)	26.0	µ Pre: 1.017 (0.010)	−0.86 (0.54)	−0.70 (0.41)	0.36
						µ Post: 1.015 (0.009)			
Somensi et al. [34]	26.1 (4.9) Recreational	Wetsuits (thickness not specified) (n = 9)	60	Florianopolis, Brazil (NR)	19.0	NR	−0.98 (0.14) ^†^ (water)	−0.70 (0.10) ^†^	0.7
							−0.63 (0.66) ^†^ (0.7 g/L Na)	−0.46 (0.47) ^†^	0.46
							−0.70 (0.84) ^†^ (0.45 g/L Na)	−0.46 (0.55) ^†^	0.46
					Overall Calculated Mean		−1.30	−0.89	0.57
					Overall Calculated Weighted Mean		−0.89	−0.73	0.54

* Calculated from pre- and post-values provided in the paper. ^†^ Interventional study involving fluid intake; µ refers to urine specific gravity, and Ò refers to urine osmolality (mOsmol/kgH_2_O). ^ꞩ^ Fluid loss was estimated based on the total fluid intake of 3 mL/kg every 20 min, using an average body mass of 73.2 kg, therefore receiving 1.2 L. NR = not reported; _NF_ = no fluid; _FI_ = fluid intake; AUS = Australia; USA = United States of America. º Refers to data that was provided directly by the authors and not extracted from the original paper. Explanatory footnote: Carrasco [17] calculated the degree of body mass loss, accounting for substrate oxidation and water metabolism (estimated at 0.415 kg). Body mass loss accounting for substrate oxidation and water metabolism for the FI group and the NF group was −0.7 (0.4) kg and −2.4 (0.6) kg, respectively; when expressed as a percentage, the losses for the FI and NF groups were −1.0 (0.5)% and −3.3 (0.7)%, respectively.

**Table 4:** Fluid balance and fluid loss across observational cross-sectional studies, stratified by the use of a wetsuit and competitive status [10,18,19,31].

Group	Author	Body Mass Change (%)	Fluid Loss Rate (L/h)	Water Temp (°C)	Duration (min)	Sample Size
		Mean (SD)	Mean		Mean (SD)	
Recreational surfers						
Wetsuit	Meir et al. [18]	−0.76 (1.00)	0.48	20	75.5 (5.0)	7
	Atencio et al. [10]	−0.60 (0.40)	0.42	24.7	119.4 (11.0)	35
	Atencio et al. [10]	−0.55 (0.57)	0.32	16.26	72.4 (30.6)	36
	Atencio et al. [10]	−0.44 (0.55)	0.26	17.19	78.4 (33.0)	60
	Average (minimum and maximum)	−0.59	0.37	19.5 (16.7 to 24.7)	86.4 (75.5 to 119.4)	Total: 138
	Weighted Average	−0.53	0.33	-	-	
No Wetsuit	Meir et al. [19]	−1.20 (1.60)	0.71	24.3	75.8 (5.9)	7
	Atencio et al. [10]	−1.15 (0.75)	0.4	25.3	121.5 (8.9)	17
	Atencio et al. [10]	−0.98 (0.78)	0.52	28.6	82.6 (34.0)	158
	O’Neil et al. [31]	−0.86 (0.54)	0.35	26	120 *	10
	Average (minimum and maximum)	−1.05	0.50	26.1 (24.4 to 28.6)	100.2 (75.8 to 120.0)	Total: 192
	Weighted Average	−1.00	0.51	-	-	
Elite Surfers						
Wetsuit	Carrasco [17]	−3.90 (0.70)	1.68	20.8	100.0 *	12

* Specified time.

**Table 5:** Confounding variables that influence fluid loss results [10,17,18,19,31,33,34].

Study	Nude Body Mass	Hair Wet	Bladder Voided Pre-Trial	Urine Loss Accounted for During Surfing	Pre-Trial Diet/ Fluid Standardisation
Atencio et al. [10]	x	x	x	NR	NR
Carrasco [17]	Semi-nude	NR	x	NR	x (provided a 1000 mL of water the night prior to the trial)
Meir et al. [19]	Semi-nude	NR	x	x	NR
Meir et al. [18]	Semi-nude	NR	x	x	x
O’Neill et al. [31]	Underwear or boardshorts	NR	x	NR	NR
Somensi et al. [34]	Bathing suits	NR	NR	NR	x (500 mL 20 min prior to trial).
Burini et al. [33]	NR	NR	NR	NR	NR

NR = Not Reported; x = confounding variable accounted for in the study

## Data Availability

The original contributions presented in this study are included in the article/Supplementary Material. Further inquiries can be directed to the corresponding author.

## Supplementary Materials

The following supporting information can be downloaded at: https://www.mdpi.com/article/10.3390/sports14020049/s1, File S1: Preferred Reporting Items for Systematic reviews and Meta-Analyses extension for Scoping Reviews (PRISMA-ScR) Checklist; File S2: Search strategies utilised for database searches and Google Scholar.
